# Hand hygiene knowledge and practices among patients in selected health facilities of Mukono and Kagadi districts in Uganda: A cross-sectional study

**DOI:** 10.1016/j.ijregi.2026.100920

**Published:** 2026-05-20

**Authors:** Fred Twinomugisha, John Bosco Isunju, Esther Buregyeya, David Musoke, Mary Nakafeero, Henry Kajumbula, Habib Yakubu, Richard K. Mugambe

**Affiliations:** 1 Department of Disease Control and Environmental Health, Makerere University School of Public Health, Kampala, Uganda; 2 Department of Epidemiology and Biostatistics, Makerere University School of Public Health, Kampala, Uganda; 3 Department of Microbiology, Makerere University School of Biomedical Sciences, Kampala, Uganda; 4 The Center for Global Safe Water, Sanitation, and Hygiene, Emory University, Rollins School of Public Health, Atlanta, GA, USA

**Keywords:** Hand hygiene, Knowledge, Patients, Mukono, Kagadi, Practice, Uganda

## Abstract

**Background:**

Hand hygiene (HH) is a cost-effective measure for preventing healthcare–associated infections, yet patient involvement in promoting HH in healthcare facilities remains underexplored in low-resource settings. This study assessed HH knowledge and practices among patients attending selected health facilities in Mukono and Kagadi districts in Uganda.

**Methods:**

A descriptive cross-sectional study was conducted between May and June 2025 among 854 adult patients from 32 purposively selected health facilities. Data were collected using an interviewer-administered structured questionnaire programmed in KoboCollect, including items on self-reported HH practices. Data were analyzed in Stata 14 using descriptive statistics and modified Poisson regression to identify factors associated with HH knowledge and practices.

**Results:**

Overall, 61.5% of respondents had adequate HH knowledge, while only 29.6% demonstrated good HH practices. Most participants (92.9%) identified infection prevention as the main purpose of HH, and 59.5% reported receiving HH information from health care workers. Adequate knowledge was higher among patients from Mukono (adjusted prevalence ratio [aPR] = 1.38; 95% confidence interval [CI]: 1.22-1.57), older participants (aPR = 1.01; 95% CI: 1.00-1.01), and those of middle socioeconomic status (aPR = 1.15; 95% CI: 1.01-1.30). However, good HH practices were less prevalent in Mukono (aPR = 0.55; 95% CI: 0.44-0.69) and among rural residents of Kagadi districts (aPR = 0.70; 95% CI: 0.56-0.88).

**Conclusion:**

Despite relatively high HH knowledge, patient practices were suboptimal. Targeted behavioral interventions are needed to bridge the knowledge–practice gap.

## Highlights

• Most patients had adequate hand hygiene (HH) knowledge, but fewer practiced it, highlighting a knowledge–practice gap.
• High awareness: 92.9% of patients recognized HH as important for infection prevention, but only 59.5% received information from health care workers (HCWs).
• Many patients were uncomfortable reminding HCWs to clean their hands.
• Limited access to handwashing facilities, soap, and sanitizers hindered proper HH practices.
• HH knowledge was higher among Mukono residents, older individuals, and groups of middle socioeconomic status.
• Good practice was associated with private facilities, female gender, and urban residence.

## Introduction

Hand hygiene (HH) is globally recognized as one of the most effective and affordable measures for preventing health care–associated infections (HAIs) and limiting the spread of infectious diseases within communities [1,2]. The World Health Organization (WHO) emphasizes that appropriate HH practices, particularly handwashing with soap or alcohol-based hand rub, can significantly reduce pathogen transmission and improve overall health outcomes [3].

While substantial efforts by many researchers have focused on improving HH compliance among health care workers (HCWs) [4,5], patients also play a critical role in infection prevention, especially in low-resource settings where overcrowding, limited sanitation facilities, and high patient mobility increase the risk of infection transmission [6,7].

Evidence shows that multimodal strategies have the greatest potential to improve HH compliance and maintain improvements in health care facilities (HCFs) [8]. The WHO Guidelines on Hand Hygiene in Health Care recommend a multimodal approach that includes system change, education, monitoring, reminders, and fostering a safety culture to increase compliance [9]. In line with these recommendations, the guidelines also endorse patient involvement in promoting HH, highlighting collaboration between patients, their families, and health care providers to enhance infection prevention practices [10]. Furthermore, the guidelines emphasize the importance of empowering patients to actively participate in their care, including supporting and reminding HCWs to perform HH when appropriate [11].

Patient knowledge and practice of HH are essential elements of infection prevention, as they influence both awareness of hygiene principles and the behaviors that reduce the transmission of pathogens [6,12]. While many patients may understand the basic importance of HH, particularly after using the toilet, before eating, or when visibly soiled, gaps often persist in their awareness of the critical moments for HH within HCF settings, such as after touching surfaces, before interacting with medical devices, or after contact with bodily fluids [12,13]. These knowledge gaps frequently translate into inconsistent practice, especially in environments where access to clean water, soap, or alcohol-based hand rub is limited [14]. In addition, cultural beliefs, competing priorities, and limited health education can reduce adherence to recommended hygiene behavior [15].

In Uganda, recurring outbreaks of infectious diseases underscore the need to strengthen infection prevention practices across health care settings and the community. These include HAIs and community-acquired infections, such as cholera and other diarrheal illnesses, as well as viral diseases such as Ebola virus disease [[16], [17], [18]]. Understanding patients’ knowledge and practices regarding HH is crucial because their behaviors can influence their risk of infection and contribute to overall infection prevention and the safety of the health care environment.

Existing evidence in Uganda on HH has largely focused on HCWs, infrastructure availability, and infection prevention and control (IPC) compliance within HCFs [19,20]. National efforts led by the Ministry of Health have emphasized the WHO “My Five Moments for HH” framework and facility-based IPC strengthening. However, limited empirical data exist on the role of patients in promoting HH practices, particularly at the facility level. While some studies have assessed HH knowledge and practices among HCWs and communities [[21], [22], [23]], there remains a paucity of district-specific evidence on patient knowledge, behavioral practices, and contextual determinants of HH within HCFs. Moreover, few studies have compared peri-urban and rural settings to explore how differences in health literacy, access to hygiene infrastructure, and sociocultural norms shape patient engagement in HH. Additionally, according to the national IPC survey conducted in Mukono and Kagadi districts, both districts reported low HH compliance among HCWs, with Mukono at 17% and Kagadi at 22% [24]. These levels are well below the WHO-recommended compliance target of at least 90% [11], emphasizing the need for further investigation to better understand the factors influencing HH practices in these settings.

This gap limits policymakers’ and facility managers’ ability to design context-specific, patient-centered interventions that complement HCW-focused IPC strategies. Therefore, this study assessed HH knowledge and practices among patients attending HCFs in the Mukono and Kagadi districts.

## Methods and materials

### Study setting

The study was conducted in two districts: Mukono and Kagadi. Mukono District is located in central Uganda, approximately 21.9 km from Kampala city. It is one of the largest districts in the country, with an estimated population of 929,224 [25]. It has a population density of approximately 179 people per km², and over 87% of the population lives in rural areas. The district has 22 clinics, 69 Health Centre (HC) IIs, 16 HC IIIs, three HC IVs, and three general hospitals [26]. Kagadi District is located in Western Uganda, approximately 255 km from Kampala city. Kagadi District has a total of 31 HCFs: 19 government, eight private for-profits (PFPs), and four private not-for-profits (PNFPs). The district has four clinics, 11 HC IIs, 14 HC IIIs, 1 HC IV, and one general hospital [26]. The district is located in the mid-western sub-region, with a total population of 471,111 [25], and is predominantly rural. Data were collected from 32 selected HCFs, comprising 16 facilities per district. Of these facilities, 21 were public, four were PFPs, and seven were PNFPs. The selected HCFs represented different levels of health care delivery and included 22 HC IIIs, three HC IVs, two clinics, and five general hospitals. Uganda’s health care system is organized in a tiered structure comprising lower-level primary care facilities and higher-level referral facilities. At the community and primary care level, health centers (e.g., HC IIs, IIIs, and IVs) provide progressively more comprehensive outpatient, preventive, and basic inpatient services. HC IVs function as lower-level hospitals with surgical capacity. General hospitals serve as first-level referral facilities, offering a broader range of inpatient and specialized services, while clinics are typically smaller, privately or publicly operated outpatient facilities providing basic curative care. The facilities included in this study were selected across these different levels to capture variation in service delivery context, patient load, and organization of care [26]. These facilities experience varying but generally substantial patient attendance, reflecting their level within the health system and catchment population.

### Study design

This study used a descriptive cross-sectional design to assess HH knowledge and practices among adult patients attending selected HCFs in Mukono and Kagadi districts in Uganda.

### Study population and eligibility criteria

The study population comprised adult patients (aged ≥18 years) receiving care in the medical, surgical, and maternity wards of selected HCFs in the Mukono and Kagadi districts in Uganda. Eligible participants were patients aged ≥18 years who were admitted to or receiving care in these wards during the data collection period. Patients who were critically ill or unable to respond to the interview at the time of data collection were excluded.

### Sample size and sampling

A total of 854 patients participated in this cross-sectional study. The sample size was determined using the Kish–Leslie formula for estimating a single population proportion [20]. The calculation assumed a 95% confidence level (Z=1.96), an estimated prevalence (p) of 50% for adequate HH knowledge/practices, and a margin of error (d) of 5%. The minimum required sample size was adjusted to account for a 10% non-response rate, yielding a final sample size of 854 participants. To ensure proportional allocation of the sample across facilities and wards and to represent the different service areas, a multistage sampling approach was used. First, HCFs in Mukono and Kagadi districts were purposively selected to represent different levels of care and service delivery contexts. Within each selected facility, the medical, surgical, and maternity wards were included in the study. These wards are high-risk areas for HAIs because of frequent patient-HCW contact, invasive procedures, wound care, and exposure to bodily fluids. HH is therefore critical in these settings, making them suitable for evaluating patient knowledge and practices [27]. Within each facility, proportional allocation was conducted across the medical, surgical, and maternity wards according to each ward’s average bed capacity or patient load, to ensure adequate representation of the different service areas. If a selected patient declined participation or did not meet the inclusion criteria, the next eligible patient was approached. Sampling within each ward continued until the required allocated sample size was attained, resulting in a total of 854 respondents. This approach enabled the study to capture a broad range of patient experiences and perspectives regarding HH knowledge and practices in the selected HCFs. Health facilities were purposively selected on the basis of their high patient volumes to ensure adequate representation of patients accessing routine medical, surgical, and maternity services.

### Data collection tools and procedures

Data were collected between May and June 2025 from adult patients attending selected HCFs in the Mukono and Kagadi districts in Uganda. A structured questionnaire, programmed and preloaded into the KoboCollect platform (Kobo Organization), was used to assess patients’ HH knowledge and self-reported practices in HCF settings. The questionnaire was developed on the basis of a review of previous studies [[27], [28]] and adapted to the local context. The tool comprised sections on sociodemographic characteristics, HH knowledge, and reported HH practices during HCF visits. Data were collected through face-to-face interviews conducted by trained research assistants, who administered the questionnaire in a private setting within the HCF premises to ensure confidentiality. Interviews were conducted in English or the local language (Luganda in Mukono District or Runyoro in Kagadi District), with English versions translated into the local language as needed, depending on the participant’s preference. The questionnaire was pretested at Kasangati HC IV, a similar HCF setting outside the study area, and necessary revisions were made to improve clarity and consistency. Each questionnaire was administered for 20-30 minutes. Completed questionnaires were checked daily by the study supervisors on the KoboCollect server to ensure completeness, accuracy, and consistency. Any identified issues were communicated to data collectors for timely correction and quality improvement.

### Quality control and assurance

Several measures were implemented to ensure the quality and reliability of the data collected. Data collection was conducted by trained research assistants with experience in health research and data collection. All research assistants held at least a bachelor’s degree, with some holding postgraduate qualifications in public health or the social sciences, and had experience administering interviewer-based questionnaires. Before data collection, the research assistants underwent a 3-day training facilitated by the researchers. The training covered the study objectives, data collection procedures, ethical considerations, informed consent, interviewing techniques, and accurate questionnaire completion. During data collection, supervisors conducted daily reviews of completed questionnaires to check for completeness, accuracy, and internal consistency. Any identified issues were addressed promptly through feedback to the research assistants. Data were securely stored in password-protected electronic databases; access was restricted to the study team to maintain confidentiality and data integrity.

### Measurement of HH knowledge and practice

Participant knowledge of HH was assessed using six structured questions adapted from previously published HH and IPC studies [29,30]. The questions covered key domains of HH knowledge relevant to patients in health care settings, including (1) the recommended duration of HH, (2) receipt of HH information from HCWs, (3) the most effective method for preventing HAIs, (4) the importance of HH in recovery from illness, (5) the role of HH in preventing infections, and (6) knowledge of appropriate HH techniques. These items were selected to capture participants’ understanding of fundamental HH concepts, perceived importance of HH, and exposure to HH education in health care settings. Each correct response was scored as 1 point, yielding a maximum possible score of 6. Participants who answered four or more questions correctly (≥67%) were categorized as having adequate HH knowledge, while those scoring <4 (<67%) were considered to have inadequate HH knowledge. Similar percentage-based cutoffs (60-70%) have been widely used in HH and knowledge, attitudes, and practices (KAP) studies to distinguish between adequate and inadequate knowledge levels [28,31,32]. Participant HH practice was assessed using three self-reported and patient-observable indicators of HH behavior and engagement in health care settings: (1) whether the participant cleaned their hands while at the HCF; (2) whether the participant felt comfortable asking a HCW to clean their hands before patient contact; and (3) whether HCWs were observed cleaning their hands before touching the participant. These indicators were included to capture different dimensions of patient involvement in HH promotion, including personal HH behavior, willingness to participate in HH advocacy, and patient observation of HCW HH practices. The “before patient contact” moment was specifically selected because it is the WHO HH moment most directly observable and most relevant to patients during care interactions [33]. We acknowledge that these measures reflect a combination of self-reported HH behavior, perceptions, and patient engagement rather than direct observation of participants’ HH practices alone. In particular, comfort with reminding HCWs may be more reflective of patient empowerment and attitudes toward participation in IPC practices. Nevertheless, these indicators were considered appropriate for assessing patient involvement in HH during health care interactions. Each appropriate response was scored 1 point, yielding a maximum score of 3. Participants scoring at least two of the three items (≥67%) were categorized as having good HH-related practice/engagement, while those scoring fewer than two were categorized as having poor HH-related practice/engagement. This threshold was used to identify participants demonstrating adherence to most of the recommended HH behaviors [34].

### Data management and analysis

Completed questionnaires were downloaded and exported to Microsoft Excel for initial cleaning and quality checks, and subsequently imported into Stata version 14 (StataCorp) for data management and statistical analysis. Categorical variables were assigned numerical codes before analysis. Composite indices were generated to measure HH knowledge and practices based on responses to relevant questionnaire items. For each domain, correct or appropriate responses were assigned a score of 1, while incorrect or inappropriate responses were assigned a score of 0. Individual scores were summed and categorized using predefined cutoff points to classify respondents as having adequate or inadequate knowledge, and good or poor HH practices**,** respectively. Patients’ self-reported socioeconomic status (SES) was assessed using a 10-step ladder scale and categorized into three levels: low (steps 1-3), middle (steps 4-6), and high (steps 7-10) [35,36]. Descriptive statistics were used to summarize participants’ characteristics, HH knowledge, and practices. Cross-tabulations were conducted to examine the distribution of outcome variables across explanatory variables. Modified Poisson regression was used to examine associations between patient HH knowledge and practices, as the outcomes had a prevalence >10%. Multicollinearity was ruled out using the variance inflation factor. A backward model-building process was used to arrive at the final model, including variables independently associated with the outcome variables at a 20% level of significance in the bivariate model. These were included in the multivariable model. The Bayesian information criterion (BIC) was also used for model selection. Adjusted prevalence ratios (aPRs) and their corresponding 95% confidence intervals (CIs) were presented to show the magnitude of the association. The Hosmer–Lemeshow test was used to assess the goodness-of-fit of the final model.

## Results

### Background characteristics of respondents

Of the respondents, 57% (n = 493) were from Mukono District, 71.4% (n = 610) attended government facilities, and most (46.8%, n = 400) were from HC IIIs; 77.6% (n = 663) were female, and the mean age was 31.5 years (SD=11.9). Most respondents had attained primary education (42.9%, n = 266), followed by secondary education (27.8%, n = 237). Most respondents (76%, n = 649) were married. Regarding religious affiliation, most were Catholic (35.8%, n = 306), followed by Anglicans (19.8%, n = 169). Only 25.8% (n = 220) were urban dwellers, while 52.55% (n = 448) reported a middle SES. Only 6.3% (n = 89) reported at least three hospitalizations in the previous year. Of the 7.8% (n = 67) who stayed at the facility for at least 4 days, most (54.2%, n = 463) were admitted to the medical ward (Table 1).

**Table 1:** Background characteristics of respondents.

Characteristics		Frequency	Percentage
		n = 854	(%)
**District**	Kagadi	361	42.3
	Mukono	493	57.7
**Facility type**			
	Government	610	71.4
	Private for-profit	131	15.3
	Private not-for-profit	113	13.2
**Facility level**			
	Clinic	26	3.0
	HC III	400	46.8
	HC IV	89	10.4
	Hospital	339	39.7
**Sex**			
	Male	191	22.4
	Female	663	77.6
**Age (years)**			
	Mean (SD)	31.5 (11.9)	
	Min, max	(18, 84)	
**Education level**			
	No education	158	18.5
	Primary	366	42.9
	Secondary	237	27.8
	Tertiary	93	10.9
**Marital status**			
	Single	132	15.5
	Married/Cohabiting	649	76.0
	Separated/Widowed	73	8.5
**Religion**			
	Catholic	306	35.8
	Muslim	110	12.9
	Pentecostal	165	19.3
	Anglican	169	19.8
	Other religion	104	12.2
**Residence**			
	Urban	220	25.8
	Rural	634	74.2
**Socioeconomic status**			
	Low	317	37.1
	Middle	448	52.5
	High	89	10.4
**Number of hospitalizations**			
	1	662	77.5
	2	138	16.2
	At least 3	54	6.3
**Length of hospital stay at the time of interview (days)**			
	1-3	769	90.0
	4-6	67	7.8
	≥1 week	18	2.1
**Ward receiving care in**			
	Maternity	337	39.5
	Medical	463	54.2
	Surgical	54	6.3

HC, Health Centre.

### Distribution of respondents’ knowledge on HH

The overall adequate knowledge rate was 61.5%, defined as correctly answering at least four of the six HH knowledge questions; 59.5% (n = 508) reported being informed about HH by HCWs. Regarding the main purpose of HH, most (92.9%, 751/854) cited infection prevention. Most respondents reported that HH should take >30 seconds (43.9%, 375/854). Most respondents (61.8%, n = 528) identified HH as the most effective method for preventing HAIs, followed by personal protective equipment (25.5%, n = 218). Respondents received HH information from multiple sources, with radio (56.7%) and medical staff (45.7%) being the most common, followed by television (36.4%). Most respondents agreed that HH is important for recovery (85.4%) and that it can prevent infection (94.4%). Knowledge of HH methods was also high: 91.1% recognized the importance of soap and 44.5% identified the need for running water (Table 2).

**Table 2:** Distribution of respondents’ knowledge on various HH aspects.

	Frequency, n = 854	Percentage (%)
**Overall HH knowledge**	525	61.5
Informed about hand hygiene by HCW		
No	346	40.5
Yes	508	59.5
Main purpose of HH		
Prevents infections	751	92.9
Professionalism	150	18.6
Protect themselves	355	43.9
Prevent spread	215	26.6
How long does one wash their hands		
<10 seconds	96	11.2
10-20 seconds	113	13.2
20-30 seconds	98	11.5
>30 seconds	375	43.9
Do not know	172	20.1
Most effective method for preventing HAIs		
Using personal protective equipment (PPE)	218	25.5
Proper HH	528	61.8
Regular cleaning of the health care environment	91	10.7
Taking antibiotics	17	2.0
Source of information on HH		
Television	311	36.4
Newspaper	45	5.3
Magazine	14	1.6
Internet	101	11.8
Medical staff	390	45.7
Radio	484	56.7
Friend	208	24.4
Megaphones	179	21.0
Other	46	5.4
Handwashing is important for recovery		
Yes	729	85.4
No	45	5.3
I do not know	80	9.4
HH prevents infection		
Yes	806	94.4
I do not know	34	4.0
No	14	1.6
Handwashing methods known		
Wash basin		
Flowing water	380	44.5
Using soap	778	91.1

HAI, health care–associated infection; HCW, health care worker; HH, hand hygiene.

### HH practices

Overall, 29.6% (253/854) of respondents demonstrated good HH-related practices and engagement. The assessment of this domain included respondents’ personal HH behavior in HCFs, their willingness to engage HCWs in HH, and their observations of HCW HH practices during care interactions. Concerning observations of HCW HH behavior, 25.3% (n = 182) of respondents reported that HCWs always cleaned their hands before touching them, while 35.4% (n = 255) stated that this occurred most of the time. Regarding patient engagement in HH promotion, 43.8% (n = 374) of respondents reported feeling comfortable asking HCWs to clean their hands before patient contact, whereas 44.0% (n = 376) reported feeling uncomfortable doing so. Among respondents who reported barriers to reminding HCWs, the most commonly cited reason was fear of offending the HCW (74.2%, n = 356), followed by shyness (26.7%, n = 128). Additionally, 12.7% (n = 61) reported uncertainty about whether it was appropriate or necessary to ask HCWs to perform HH. Regarding respondents’ personal HH behaviors within HCFs, 35.0% (n = 299) reported frequently cleaning their hands, whereas 10.4% (n = 89) reported not cleaning their hands at all during their visit. Among those who did not clean their hands (n = 89), the most commonly reported reason was forgetting (32.6%, n = 29), followed by the perception that HH was not necessary (25.8%, n = 23). Structural barriers affecting HH practices were also identified, including lack of soap or sanitizer (21.3%, n = 19) and absence of handwashing stations (15.7%, n = 14) (Table 3).

**Table 3:** Description of respondents’ HH practices.

	Frequency	Percentage
	N = 854	(%)
**Overall practice** 253 29.6 Health care workers are observed cleaning their hands before touching the patient		
Always	182	25.3
Most of the time	255	35.4
Never	2	0.3
Rarely	41	5.7
Sometimes	240	33.3
The participant feels comfortable asking a health care worker to clean their hands before patient contact		
No	376	44.0
Sometimes	104	12.2
Yes	374	43.8
Barriers that prevent the participant from asking a health care worker to clean their hands before patient contact		
Feel shy	128	26.7
Not to offend	356	74.2
Not sure if it is necessary	61	12.7
Other	14	2.9
The participant cleans their hands while at the health care facility		
Yes, frequently	299	35.0
Sometimes	466	54.6
No	89	10.4
Reasons for not cleaning hands		
I do not think it is necessary	23	25.8
I forget	29	32.6
No hand washing stations	14	15.7
No soap or sanitizer available	19	21.3
Others (Specify)	4	4.5

HH, hand hygiene.

### Factors associated with adequate knowledge

The multivariable modified Poisson regression model assessing factors associated with adequate HH knowledge demonstrated a good fit to the data, as indicated by a Hosmer–Lemeshow goodness-of-fit p-value of 0.983. The final model was further supported by the lowest BIC value of 1592.3. The prevalence of adequate HH knowledge was significantly higher among patients from Mukono than those from Kagadi District (aPR = 1.38; 95% CI: 1.22-1.57; *P* < 0.001). Increasing age was also independently associated with adequate HH knowledge, with a 1% increase in prevalence for each additional year of age (aPR = 1.01; 95% CI: 1.00-1.01; *P* = 0.034). SES was another significant predictor. Compared with patients in the low-SES category, those in the middle-SES category had a 15% higher prevalence of adequate HH knowledge (aPR = 1.15; 95% CI: 1.01-1.30; *P* = 0.029). No significant association was observed among patients in the high-SES category. Length of hospital stay at the time of interview was inversely associated with adequate HH knowledge. Patients who had stayed in the health facility for 4-6 days had a 23% lower prevalence of adequate HH knowledge compared with those who had stayed for 1-3 days (aPR = 0.77; 95% CI: 0.61-0.96; *P* = 0.022). A stay of ≥1 week was not significantly associated with HH knowledge. Other factors, including sex, facility ownership, facility level, education level, marital status, religion, residence, number of recent hospitalizations, duration of care at the facility, and ward of admission, were not independently associated with adequate HH knowledge in the adjusted analysis (Table 4).

**Table 4:** Modified Poisson regression for factors associated with possession of adequate HH knowledge among patients in Mukono and Kagadi districts.

	Adequate knowledge		Crude PR (95% CI)	Adjusted PR (95% CI)	p-value
	N	Freq (row%)			
	Overall	854	**525 (61.5)**		
**District**					
Kagadi	361	177 (49.0)	1	1	
Mukono	493	348 (70.6)	1.44 (1.28-1.62)	1.38 (1.22-1.57)	<0.001
**Facility type**					
Government	610	374 (61.3)	1		
Private for-profit	131	82 (62.6)	1.02 (0.88-1.18)		
Private not-for-profit	113	69 (61.1)	0.99 (0.85-1.17)		
**Facility level**					
Clinic	26	15 (57.7)	1		
HC III	400	251 (62.7)	1.09 (0.78-1.53)		
HC IV	89	63 (70.8)	1.23 (0.86-1.75)		
Hospital	339	196 (57.8)	1.00 (0.71-1.41)		
**Sex of respondent**					
Male	191	115 (60.2)	1	1	
Female	663	410 (61.8)	1.03 (0.90-1.17)	1.12 (0.98-1.28)	0.092
**Age (years)**	854	31.5 (11.0)	1.00 (0.99-1.01)	1.01 (1.00-1.01)	0.034
**Education level**					
No education	158	92 (58.2)	1		
Primary	366	214 (58.5)	1.00 (0.86-1.76)		
Secondary	237	149 (62.9)	1.08 (0.92-1.27)		
Tertiary	93	70 (75.3)	1.29 (1.08-1.54)		
**Marital status**					
Single	132	77 (58.3)	1		
Married/Cohabiting	649	403 (62.1)	1.07 (0.91-1.25)		
Separated/Widowed	73	45 (61.6)	1.06 (0.84-1.33)		
**Religion of the respondent**					
Catholic	306	172 (56.2)	1		
Muslim	110	75 (68.2)	1.21 (1.03-1.43)		
Pentecostal	165	103 (62.4)	1.11 (0.95-1.29)		
Anglican	169	115 (68.0)	1.21 (1.05-1.39)		
Other religion	104	60 (57.7)	1.03 (0.85-1.24)		
**Residence**					
Urban	220	149 (67.7)	1		
Rural	634	376 (59.3)	0.88 (0.78-0.98)		
**Socioeconomic status**					
Low	317	171 (53.9)	1	1	
Middle	448	307 (68.5)	1.27 (1.13-1.43)	1.15 (1.01-1.30)	0.029
High	89	47 (52.8)	0.98 (0.78-1.22)	1.00 (0.82-1.24)	0.976
**Number of hospitalizations in the previous month**					
1	662	411 (62.1)	1		
2	138	82 (59.4)	0.96 (0.82-1.11)		
≥3	54	32 (59.3)	0.96 (0.76-1.20)		
**Length of hospital stay at the time of interview**					
1-3 days	769	481 (62.5)	1	1	
4-6 days	67	35 (52.2)	0.84 (0.66-1.06)	0.77 (0.61-0.67)	0.022
≥1 week	18	9 (50.0)	0.79 (0.50-1.27)	0.78 (0.48-1.27)	0.322
**How long have you been receiving care at this facility**					
<1 week	611	370 (60.6)	1		
1-2 weeks	58	33 (56.9)	0.94 (0.74-1.19)		
>2 weeks	51	31 (60.8)	1.00 (0.79-1.26)		
For the last 9 months	134	91 (67.9)	1.12 (0.98-1.28)		
**Ward receiving care in**					
Maternity	337	199 (59.1)	1		
Medical	463	292 (63.1)	1.07 (0.95-1.19)		
Surgical	54	34 (63.0)	1.07 (0.85-1.33)		

CI, confidence interval; HC, Health Centre; HH, hand hygiene; PR, prevalence ratio.

### Factors associated with good HH practices

The multivariable modified Poisson regression model assessing factors associated with good HH practices showed a good fit to the data (Hosmer–Lemeshow *P* = 0.320) and had the lowest BIC value of 1168.8, indicating optimal model selection. The prevalence of good HH practices was significantly lower among patients from Mukono than those from Kagadi District (aPR = 0.55; 95% CI: 0.44-0.69; p<0.001). Conversely, patients attending private, for-profit facilities had a 71% higher prevalence of good practices than those attending government facilities (aPR = 1.71; 95% CI: 1.32-2.22; p<0.001). Female patients were more likely to practice good HH than males (aPR = 1.40; 95% CI: 1.04-1.89; *P* = 0.025). In terms of marital status, patients who were separated or widowed demonstrated a higher prevalence of good practices compared with never-married patients (aPR = 1.56; 95% CI: 1.09-2.21; *P* = 0.013). Residence was also associated with HH practices. Patients residing in rural areas had a 30% lower prevalence of good HH practices compared with urban residents (aPR = 0.70; 95% CI: 0.56-0.88; *P* = 0.002). SES influenced practices, with patients in the middle-SES group showing a higher prevalence of good HH practices than those in the low-SES group (aPR = 1.33; 95% CI: 1.05-1.69; *P* = 0.019). Hospitalization history also affected HH. Patients who had been hospitalized at least three times in the past month had a lower prevalence of good HH practices than those hospitalized once (aPR = 0.49; 95% CI: 0.27-0.91; *P* = 0.023). Similarly, patients admitted to medical wards had a lower prevalence of good HH practices than those in maternity wards (aPR = 0.79; 95% CI: 0.63-0.99; *P* = 0.044). Other variables, including age, education level, religion, facility level, and length of hospital stay, were not independently associated with good HH practices in the adjusted analysis (Table 5).

**Table 5:** Modified Poisson regression for factors associated with good HH practices among patients in Mukono and Kagadi districts.

	Adequate practice		Crude PR (95% CI)	Adjusted PR (95% CI)	p-value
	N	Freq (row%)			
Overall	**854**	**253 (29.6)**			
**District**					
Kagadi	361	128 (35.5)	1	1	
Mukono	493	125 (25.4)	0.72 (0.58-0.88)	0.55 (0.44-0.69)	<0.001
**Facility type**					
Government	610	172 (28.2)	1	1	
Private for-profit	131	53 (40.5)	1.43 (1.12-1.83)	1.71 (1.32-2.22)	<0.001
Private not-for-profit	113	28 (24.8)	0.88 (0.62-1.24)	0.91 (0.66-1.26)	0.572
**Facility level**					
Clinic	26	12 (46.2)	1		
HC III	400	116 (29.0)	0.63 (0.40-0.98)		
HC IV	89	14 (15.7)	0.34 (0.18-0.64)		
Hospital	339	111 (32.7)	0.71 (0.46-1.10)		
**Sex of respondent**					
Male	191	44 (23.0)	1		
Female	663	209 (31.5)	1.36 (1.03-1.82)	1.40 (1.04-1.89)	0.025
**Age (years), Mean (SD)**	854	31.5 (11.9)	0.99 (0.98-1.01)		
**Education level**					
No education	158	45 (28.5)	1		
Primary	366	108 (29.5)	1.04 (0.77-1.39)		
Secondary	237	65 (27.4)	0.96 (0.69-1.33)		
Tertiary	93	35 (37.6)	1.32 (0.92-1.89)		
**Marital status**					
Single	132	39 (29.5)	1	1	
Married/Cohabiting	649	181 (27.9)	0.94 (0.71-1.26)	0.84 (0.63-1.13)	0245
Separated/Widowed	73	33 (45.2)	1.53 (1.06-2.20)	1.56 (1.09-2.21)	0.013
**Religion of the respondent**					
Catholic	306	97 (31.7)	1		
Muslim	110	28 (25.5)	0.80 (0.56-1.15)		
Pentecostal	165	43 (26.1)	0.82 (0.61-1.12)		
Anglican	169	44 (26.0)	0.82 (0.61-1.11)		
Other religion	104	41 (39.4)	1.24 (0.93-1.66)		
**Residence**					
Urban	220	81 (36.8)	1	1	
Rural	634	172 (27.1)	0.74 (0.59-0.91)	0.70 (0.56-0.88)	0.002
**Socioeconomic status**					
Low	317	85 (26.8)	1	1	
Middle	448	135 (30.1)	1.12 (0.89-1.42)	1.33 (1.05-1.69)	0.019
High	89	33 (37.1)	1.38 (0.99-1.92)	1.39 (1.00-1.95)	0.050
**Number of hospitalizations in the last month**					
1	662	200 (30.2)	1	1	
2	138	44 (31.9)	1.06 (0.81-1.38)	1.01 (0.76-1.33)	0.949
≥3	54	9 (16.7)	0.55 (0.30-1.01)	0.49 (0.27-0.91)	0.023
**Length of hospital stay at the time of interview**					
1-3 days	769	235 (30.6)	1		
4-6 days	67	14 (20.9)	0.68 (0.42-1.10)		
≥1 week	18	4 (22.2)	0.73 (0.30-1.74)		
**How long have you been receiving care at the facility**					
<1 week	611	183 (30.0)	1		
1-2 weeks	58	20 (34.5)	1.15 (0.79-1.68)		
>2 weeks	51	11 (21.6)	0.72 (0.42-1.23)		
For the last 9 months	134	39 (29.1)	0.97 (0.73-1.29)		
**Ward receiving care in**					
Maternity	337	113 (33.5)	1	1	
Medical	463	128 (27.6)	0.83 (0.67-1.02)	0.79 (0.63-0.99)	0.044
Surgical	54	12 (22.2)	0.66 (0.39-1.12)	0.77 (0.45-1.33)	0.350

CI, confidence interval; HC, Health Centre; HH, hand hygiene; PR, prevalence ratio.

## Discussion

This study assessed HH knowledge and practices among patients in selected HCFs in the Mukono and Kagadi districts in Uganda. Overall, the findings indicate that although most patients (61.5%) had adequate HH knowledge, fewer than one-third (29.6%) demonstrated good HH practices within HCFs. This gap between knowledge and practice underscores the challenge of translating knowledge into consistent HH practice in health care settings. The finding that 59.5% of participants reported being informed about HH by HCWs underscores the important role of HCF providers as sources of infection-prevention information. However, the fact that over 40% had not received this information indicates missed opportunities for patient education during care delivery. These findings suggest that improving patient HH in HCF settings requires not only strengthening knowledge dissemination but also implementing structured, system-level, and behavioral interventions to address contextual barriers to practice.

The prevalence of adequate HH knowledge was significantly higher among patients in Mukono than those in Kagadi, potentially reflecting differences in exposure to structured health education, the intensity of health promotion activities, and the institutional prioritization of infection prevention. Mukono has previously benefited from targeted HH improvement initiatives, including multifaceted interventions by Mugambe *et al.* [37,38] that incorporated environmental cues, provision of alcohol-based hand rub and soap, and structured health education for HCWs and communities. Such sustained investments likely created an enabling environment in which HH messaging is reinforced at both the facility and community levels. In addition, this variation may be partly explained by ongoing national IPC strengthening efforts, including the Ministry of Health mentorship model, which provides supportive supervision and on-site coaching to improve HCW adherence to IPC and HH practices. However, the extent of implementation may vary across districts and facilities, potentially contributing to differences in patients’ exposure to HH messaging and reinforcement.

Beyond merely reflecting prior interventions, the observed district variation may indicate that knowledge acquisition is strongly influenced by contextual health system factors, including leadership commitment, resource availability, and program continuity. These findings align with broader evidence showing that comprehensive health education strategies improve infection-prevention behaviors [39,40]. Similarly, these findings underscore the importance of sustained, district-level investment in health education and infection prevention programs to reduce knowledge disparities and ultimately minimize the burden of HAIs across different settings.

Knowledge regarding the purpose of HH was notably high, with over 90% of respondents correctly identifying infection prevention as the main reason. Similarly, most respondents recognized HH as important for recovery from illness, particularly infectious diseases, and effective in preventing infections, reflecting a strong conceptual understanding of HH benefits. These findings suggest that core messages around infection prevention have penetrated the patient population. Similarly, a systematic review by Hammoud *et al.* [41] recommended that hospitals emphasize the importance of patient engagement and education on infection control, and encourage patients to involve themselves in their care by asking HCWs to provide them with information. In this way, their understanding of the importance of HH would increase, ultimately leading to a reduction in HAIs. Similar results have been reported elsewhere, with researchers emphasizing patient education [[42], [43], [44]]. These findings suggest that a high level of awareness presents a critical opportunity to engage informed patients as active partners in infection prevention efforts, thereby enhancing compliance, improving patient safety, and reducing the burden of HAIs at both the facility and community levels.

This study identified gaps in practical and procedural knowledge. Fewer than half of the respondents correctly identified the recommended handwashing duration of >30 seconds, and approximately one in five did not know the appropriate duration at all. Adequate duration is critical for effective removal of transient microorganisms; therefore, uncertainty in this area may compromise the effectiveness of otherwise well-intentioned handwashing practices. In addition, although most participants recognized soap and running water as essential components of HH, very few mentioned wash basins or broader infrastructure, indicating limited awareness of what constitutes an appropriate and functional HH facility. This limited understanding may hinder patients’ optimal use of available infrastructure and reduce their ability to assess whether facilities meet basic hygiene standards, as highlighted in previous research [45,46]. Similar gaps in practical HH knowledge among patients have been reported by Chok [33] and Lee *et al.* [47], suggesting that this is not an isolated finding but a broader challenge in patient education. From a public health perspective, these deficiencies in procedural knowledge underscore the need for targeted, practical HH education that goes beyond general messaging to include demonstrations and clear guidance on correct techniques and facility use, thereby strengthening infection prevention efforts and enhancing patient safety.

Good HH practices were significantly less common among patients in Mukono despite their higher levels of knowledge, underscoring the well-documented disconnect between knowledge and behavior [48,49]. While patients in Mukono demonstrated significantly higher adequate HH knowledge (aPR=1.38), they were substantially less likely to practice good HH (aPR=0.55). This paradox suggests that knowledge alone is insufficient to drive behavioral change [50,51]. Structural constraints such as overcrowding, higher patient volumes, longer waiting times, and strained WASH (water, sanitation, and hygiene) infrastructure in peri-urban settings like Mukono may limit the translation of knowledge into consistent practice [52,53]. In high-volume facilities, access to functional handwashing stations, soap, or alcohol-based hand rub may be inconsistent, discouraging adherence [54]. Additionally, some studies have documented over-reporting of HH knowledge in self-reported surveys, in which respondents provide socially desirable responses rather than reflecting their actual understanding or behavior [55,56]. This may partly explain the observed discrepancy between knowledge and practice.

Patients attending private for-profit facilities were significantly more likely to demonstrate good HH practices (aPR=1.71). This finding may reflect better infrastructure, more reliable availability of HH supplies, lower patient-to-provider ratios, and stronger enforcement of IPC standards [57]. Private facilities may also have stronger accountability mechanisms and customer-service cultures that encourage visible compliance with hygiene standards [58]. Female respondents were more likely to practice good HH (aPR=1.40), aligning with extensive literature indicating that women are generally more engaged in preventive health behaviors and hygiene practices [59,60]. Gender socialization patterns, caregiving roles, and differences in risk perception may contribute to this disparity.

Rural residence was independently associated with poorer HH practices (aPR=0.70), suggesting that place of residence plays a critical role in shaping patient behavior in HCF settings. Individuals from rural areas may have had less prior exposure to structured HCF environments where formal HH norms are routinely modeled and reinforced. In addition, rural communities often face persistent challenges related to water access, sanitation infrastructure, and limited availability of hygiene supplies, which can lead to the normalization of suboptimal hygiene practices over time [61,62]. These contextual disadvantages may influence both habitual behaviors and expectations regarding hygiene standards when patients seek care. Furthermore, infrastructural gaps within rural health facilities, including inconsistent water supply, inadequate handwashing stations, and limited availability of soap or alcohol-based hand rub, can further constrain patients’ ability to practice HH even when they are aware of its importance [14,63]. The interplay between community-level exposure and facility-level constraints likely contributes to the observed disparity, indicating that improving HH practices requires addressing both behavioral and structural determinants. These findings highlight the need for context-specific strategies that strengthen infrastructure in rural settings and standardize IPC promotion across all hospital wards to reduce inequities in HH practice and enhance patient safety outcomes.

Admission to medical wards was also associated with lower HH practice compared with admission to maternity wards (aPR=0.79). Maternity settings often operate within a heightened infection prevention culture because mothers and neonates are particularly vulnerable to sepsis and other HAIs [64,65]. As a result, these wards may feature more visible HH reminders, stricter supervision, closer patient–provider interactions, and stronger reinforcement of hygiene behaviors [64]. In contrast, medical wards may not consistently maintain the same intensity of infection prevention messaging or perceived urgency, potentially leading to lower patient engagement in HH practices. Although surgical wards are also recognized as high-risk settings for HAIs, particularly surgical site infections, they were not highlighted in this comparison because the observed differences in HH practices were not statistically significant relative to the reference category. Nonetheless, surgical units remain critical areas for strengthening IPC practices, and future studies should further explore patient HH behaviors and IPC culture in these settings to inform targeted improvement strategies.

### Strengths and limitations

This study provides a comprehensive assessment of HH knowledge and practices among patients across multiple facility types and regions, using robust sampling and regression analyses. However, reliance on self-reported data for HH practices may introduce social desirability bias. Furthermore, the cross-sectional design limits causal inference regarding the relationship between identified factors and HH outcomes.

## Conclusion

Although patient knowledge of HH was relatively high, actual practice remained low. Sociodemographic characteristics and facility type significantly influenced HH adherence. Sociodemographic and contextual factors, such as district, SES, sex, residence, and type of health facility, were significantly associated with both knowledge and practice. These findings highlight a critical need for targeted interventions that extend beyond awareness-raising to address behavioral, social, and structural barriers.

## CRediT authorship contribution statement

**Fred Twinomugisha:** Conceptualization, Data curation, Formal analysis, Investigation, Methodology, Project administration, Software, Supervision, Validation, Visualization, Writing – original draft, Writing – review & editing. **John Bosco Isunju:** Formal analysis, Investigation, Methodology, Resources, Supervision, Validation, Visualization, Writing – original draft, Writing – review & editing. **Esther Buregyeya:** Data curation, Funding acquisition, Investigation, Resources, Software, Supervision, Validation, Visualization, Writing – original draft, Writing – review & editing. **David Musoke:** Data curation, Investigation, Methodology, Resources, Software, Supervision, Validation, Visualization, Writing – original draft, Writing – review & editing. **Mary Nakafeero:** Data curation, Formal analysis, Investigation, Project administration, Resources, Software, Validation, Writing – original draft, Writing – review & editing. **Henry Kajumbula:** Data curation, Investigation, Methodology, Resources, Supervision, Validation, Visualization, Writing – original draft, Writing – review & editing. **Habib Yakubu:** Funding acquisition, Investigation, Methodology, Resources, Supervision, Validation, Visualization, Writing – original draft, Writing – review & editing. **Richard K. Mugambe:** Funding acquisition, Investigation, Methodology, Resources, Supervision, Validation, Visualization, Writing – original draft, Writing – review & editing.

## Declaration of competing interest

The authors have no competing interests to declare.
